# Electrospun Chitosan/Polylactic Acid Nanofibers with Silver Nanoparticles: Structure, Antibacterial, and Cytotoxic Properties

**DOI:** 10.1021/acsabm.4c01252

**Published:** 2025-01-15

**Authors:** Yevhen Samokhin, Yuliia Varava, Kateryna Diedkova, Ilya Yanko, Valeriia Korniienko, Yevheniia Husak, Igor Iatsunskyi, Vladlens Grebnevs, Maris Bertiņs, Rafal Banasiuk, Viktoriia Korniienko, Agne Ramanaviciute, Maksym Pogorielov, Arunas Ramanavicius

**Affiliations:** 1 Biomedical Research Centre, Sumy State University, 116, Kharkivska, 40007 Sumy, Ukraine; 2 Institute of Atomic Physics and Spectroscopy, University of Latvia, Jelgavas iela 3, LV-1004 Riga, Latvia; 3 Faculty of Chemistry, Silesian University of Technology, 44-100 Gliwice, Poland; 4 NanoBioMedical Centre, Adam Mickiewicz University, Wszechnicy Piastowskiej 3, 61-614 Poznan, Poland; 5 Faculty of Chemistry, University of Latvia, Jelgavas iela 1, LV-1004 Riga, Latvia; 6 NanoWave, 02-676 Warsaw, Poland; 7 Department of Physical Chemistry, Institute of Chemistry, Faculty of Chemistry and Geosciences, Vilnius University, Naugarduko Str. 24, LT-03225 Vilnius, Lithuania

**Keywords:** chitosan, polylactic acid, electrospinning, nanofibers, biocompatibility, antibacterial activity

## Abstract

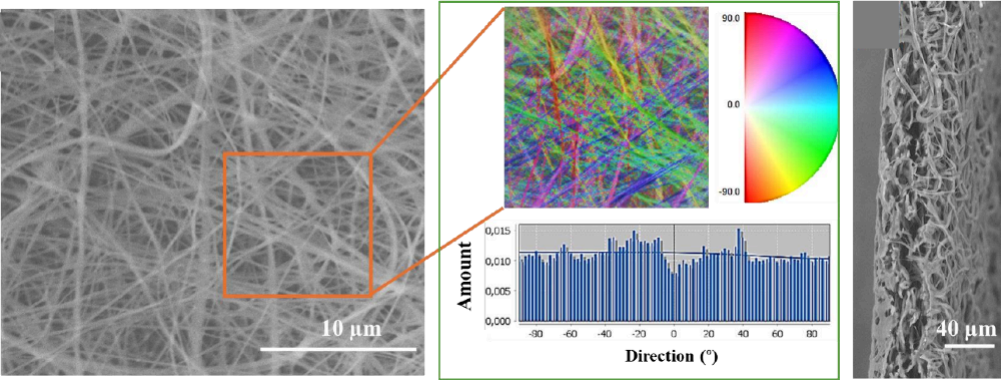

Electrospinning, a technique for creating fabric materials from polymer solutions, is widely used in various fields, including biomedicine. The unique properties of electrospun fibrous membranes, such as large surface area, compositional versatility, and customizable porous structure, make them ideal for advanced biomedical applications like tissue engineering and wound healing. By considering the high biocompatibility and well-known regenerative potential of polylactic acid (PLA) and chitosan (CH), as well as the versatile antibacterial effect of silver nanoparticles (AgNPs), this study explores the antibacterial efficacy, adhesive properties, and cytotoxicity of electrospun chitosan membranes with a unique nanofibrous structure and varying concentrations of AgNPs. Silver nanoparticles incorporated at concentrations of 25–50 μg/mL or above significantly enhanced the antibacterial effectiveness, especially against *Staphylococcus aureus* and *Escherichia coli*. Biocompatibility assessments using umbilical cord mesenchymal stem cells demonstrated the nontoxic nature of the membranes with an AgNP concentration of 12.5 μg/mL, underscoring their potential for biomedical applications. This study provides valuable insights into developing electrospun chitosan membranes as effective antimicrobial coatings for various biomedical uses, including wound healing patches and tissue engineering constructs for soft tissue replacement.

## Introduction

Electrospinning is an exceptionally versatile technique for generating continuous fibers (with diameters spanning from a few nanometers to micrometers), derived from polymer solutions.^1^ Interest and the scale of development of electrospun fibrous membranes has been growing due to their distinctive features such as a remarkably high surface area to volume ratio, extensive compositional and morphological versatility, customizable porous structure, and the ability to conform to diverse sizes and shapes. Materials created using this method have found applications across a wide spectrum, including water and air treatment, catalysis, energy, photonics, electronics, smart materials, and, most notably, in the biomedical area.^2^

Chitosan, a biopolymer derived from chitin, is recognized for its biocompatibility, biodegradability, and nontoxicity, making it an ideal candidate for various biomedical applications. Its unique properties, including antimicrobial activity, promote cell adhesion and proliferation, which are critical for tissue engineering and regenerative medicine. The extensive surface area and porous structure of electrospun chitosan fibers facilitate nutrient and gas exchange, essential for cell survival and tissue integration.^3^ Furthermore, chitosan’s ability to form hydrogels enhances its functionality in drug delivery systems, allowing for controlled release and improved therapeutic efficacy.^4^

Polylactic acid (PLA), on the other hand, is a biodegradable polyester derived from renewable resources such as corn starch or sugar cane. PLA exhibits favorable mechanical properties and is widely used in the medical field due to its excellent biocompatibility and ability to mimic the extracellular matrix (ECM).^5^ These attributes make PLA particularly suitable for applications in tissue engineering, where it can support cell growth and differentiation. The versatility of PLA allows for the modification of its physical and chemical properties, enabling the development of tailored scaffolds for specific medical needs.^6^ Together, chitosan and PLA offer complementary advantages, making them promising materials for enhancing the performance of electrospun membranes in biomedical applications.

The unique combination of attributes of electrospun matrices have sparked significant interest in the realm of biomaterials and biomedical devices.^7^ These matrices often prove well-suited to address the intricate demands of advanced applications such as tissue engineering,^8^ drug delivery,^9^ wound dressing,^10^ and enzyme immobilization.^11^ Polylactic acid (PLA) and chitosan (CH) are highly valued biomaterials due to their exceptional biocompatibility and biodegradability, making them ideal for biomedical applications, especially in medicine and tissue engineering. PLA in particular is favored for its balanced properties, closely resembling the extracellular matrix (ECM) and enabling efficient drug delivery. Electrospun chitosan biomaterials show potential for application in tissue engineering and regenerative medicine due to their extensive surface area, pore distribution, and antibacterial properties. As these materials mimic the ECM they facilitate cell processes that are critical for tissue regeneration.^12,13^

In addition to its biocompatibility and biodegradability, chitosan exhibits notable antibacterial and antibiofilm properties, making it an attractive material for biomedical applications. Numerous studies have demonstrated that chitosan can inhibit the growth of a wide range of bacteria, including both Gram-positive and Gram-negative strains.^14,15^ Its antimicrobial activity is attributed to several mechanisms, including disruption of the bacterial cell membrane and interference with metabolic processes.^16,17^ Furthermore, chitosan has been shown to effectively prevent biofilm formation and disrupt existing biofilms, making it a promising candidate for use in coatings and wound dressings to combat infections.

Given the ongoing significance of microbial infections for the public health, antibacterial agents and antibiotics have assumed a pivotal role in addressing these health concerns.^18,19^ The evolution of multidrug-resistant microorganisms underscores the pressing need for innovative solutions.^20^ A wealth of literature studies has highlighted the successful development of electrospun mats utilizing biocompatible and often biodegradable polymers.^21^ These mats exhibit the capacity to release various antibacterial agents at controlled rates. Notably, the well-established antimicrobial properties of silver and its ions have sparked numerous research endeavors focused on crafting polymeric scaffolds containing silver nanoparticles (AgNPs).^22^ These scaffolds aim to immobilize AgNPs, thereby facilitating a sustained antibacterial effect.^23^ AgNPs demonstrate enhanced capacity and a higher surface area to volume ratio compared to bulk silver.^24^ At the nanoscale, AgNPs display unique electrical, optical, and catalytic properties, prompting the exploration and development of products for targeted drug delivery, diagnosis, detection, and imaging.^25^ Moreover, AgNPs exhibit significant antimicrobial activity against various infectious and pathogenic microorganisms,^26,27^ including those resistant to multiple drugs.^28^ As a result, the remarkable antibacterial efficacy displayed by AgNPs has captured the interest of researchers and industries.^29^ The integration of AgNPs into numerous products has been explored, including surgical and food-handling tools, apparel, cosmetics, dental items, catheters, and dressings.^30^ The antibiotic potential of AgNPs stems from their diverse mechanisms of action, targeting microorganisms across multiple structures simultaneously and thus providing them the capability to combat various types of bacteria.^31,32^

One of the approaches how nanofibrous materials can be enhanced is through en-capsulation/loading with metal-based nanoparticles to amplify the therapeutic outcomes in antibacterial applications.^33,34^ Silver nanoparticles stand out among the extensively investigated metal-based nanoparticles due to their commendable properties.^35^ In addition to the aforementioned exceptional antibacterial properties, they also demonstrate antioxidant and anti-inflammatory characteristics, and facilitate cell growth, positioning them as crucial bioactive agents for antibacterial usage.^36^ Several pieces of research comprehensively present the biological outcomes (both in vivo and in vitro) as well as the mechanical effects of nanofibrous scaffolds loaded with silver nanoparticles on the wound healing process.^37^

In the current landscape, the pursuit of new antibiotics presents great challenges, necessitating extensive research periods to evaluate the effectiveness and safety of the potential agents. This process consumes substantial time and resources, all while infections caused by multiresistant microorganisms persistently increase. In the defined postantibiotic era, AgNPs, in conjunction with other nanomaterials, have been under scrutiny to identify novel agents capable of combating pathogenic microorganisms without fostering the emergence of new resistances.^38^ Given the worldwide apprehension regarding infections caused by antibiotic-resistant microorganisms, AgNPs emerge as a promising alternative. Their applications can extend to preventing infections caused by these microorganisms, decontaminating medical supplies, and even addressing ongoing infections.^39^ As a substitute for antibiotics, this avenue of exploration has undergone extensive research in recent years, with the goal of developing new bactericidal products for decontamination or infection treatments, leveraging the well-established knowledge of their efficacy, even against multidrug-resistant organisms.^40,41^

Considering the challenges in developing biocompatible membranes with advanced antibacterial properties, this study explores novel electrospun chitosan membranes with detailed analyses of their effectiveness against bacteria, adhesive characteristics, and potential cytotoxicity. By employing a blend of traditional and contemporary methodologies, this research aims to provide a comprehensive understanding of the material’s potential as an antimicrobial coating.

## Results

### Scanning Electron Microscopy (SEM) and Contact Angle

**Figure 1:**
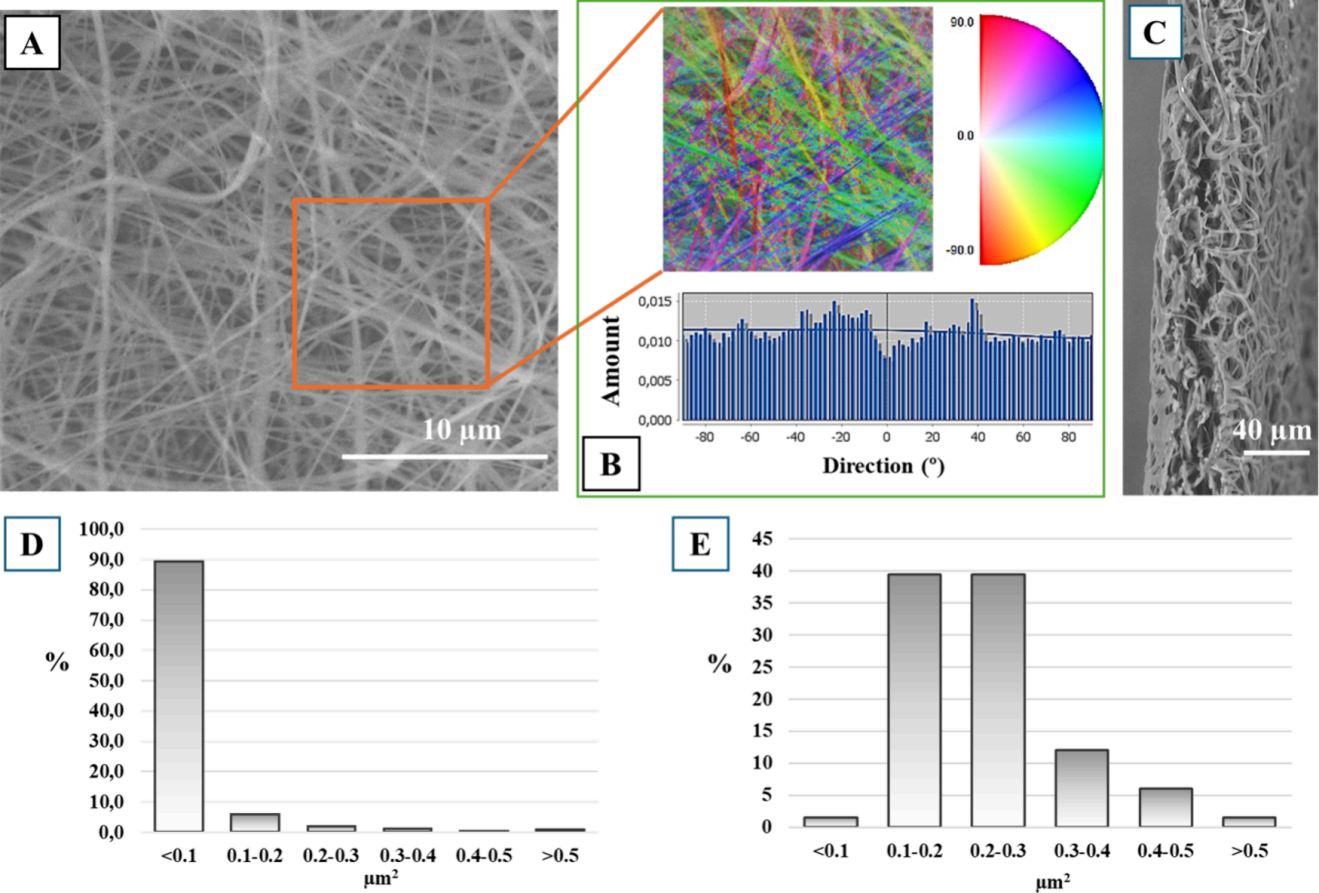
Scanning electron microscopy of CH/PLA membrane (A) with fiber orientation map and directionality histograms (B) indicating the number of fibers in a given direction, the cross-sectional view (C) of the membrane, pore size (D), and fiber diameter distribution (E).

The electrospun membrane has a high surface area with a porosity of up to 16% and a small pore size of 0.04 ± 0.01 μm^2^ (Figure 1). The fabricated fibers exhibit a random orientation pattern with bead-free morphology. The average fiber diameter in the scaffold was 0.24 ± 0.1 μm with diameters ranging from 70 to 700 nm. The nanometer’s fibers afford impeccable characteristics such as more significant surface area to volume ratio, increased flexibility toward surface modifications and functionalities, and superior mechanical performance (including stiffness and tensile strength). These properties make polymeric nanofibers ideal candidates for mimicking the extracellular matrix structure.^42^ The cross-section confirmed the porous and multilayer structure of the electrospun membrane that allows gas permeability and water sorption. Wettability is a crucial parameter for the membrane, giving an idea of the interactions between the membrane surface and the organism’s environment in vivo. It significantly influences cell adhesion, proliferation, and membrane degradation.^43^ Moreover, nanofibre membrane permeability is an important physical property, primarily when the membranes are intended for biomedical use in tissue regeneration (e.g., wound healing and tissue reconstruction).^44^ The obtained electrospun CH/PLA membranes have a hydrophilic contact angle value of 58 ± 2° with a reduction to 0° after NaOH treatment (SI, Figure S1). All of this data showcased that the material is suitable for in vivo applications as scaffold for tissue regeneration and wound healing.

**Figure 2:**
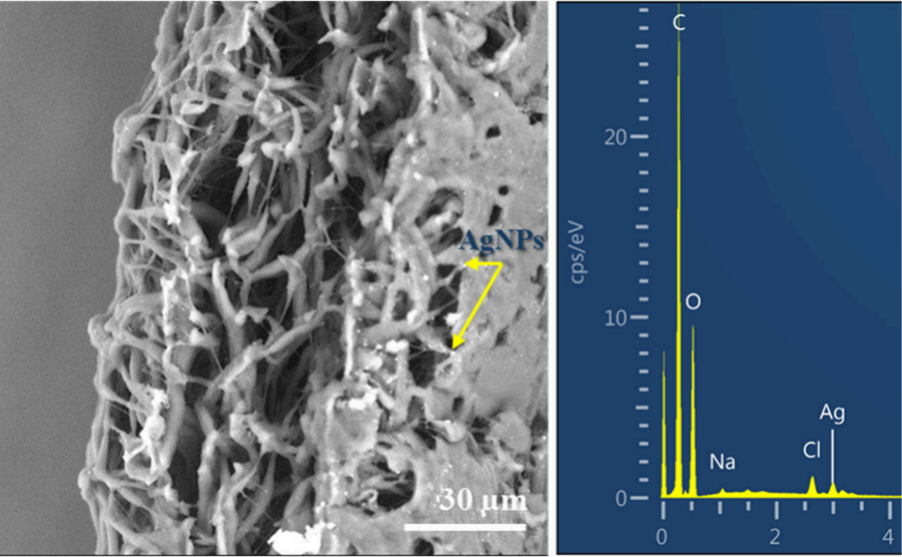
Scanning electron microscopy images of membranes’ cross-section with EDX analysis (region analysis) of membranes loaded with 400 μg/mL of AgNPs.

The decoration with AgNPs did not adversely affect the membrane’s surface morphology (Figure 2). EDX analysis confirmed the presence of silver following the particle application. The highest silver concentration of 0.25 atomic% was found in the CH/PLA 400 μg/mL AgNPs membrane.

### FTIR Spectroscopy Analysis

**Figure 3:**
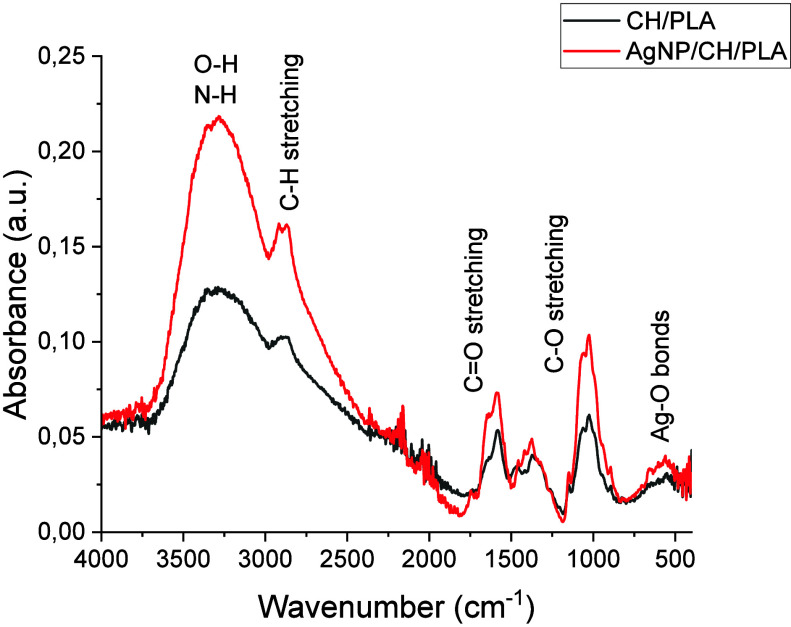
FTIR spectra of CH/PLA and CH/PLA nanofibers modified by silver nanoparticles.

The FTIR spectra of CH/PLA and CH/PLA nanofibers modified by silver nanoparticles reveal distinct vibrational signatures characteristic of the composite materials (Figure 3). In both spectra, a broad peak in the 3000–3600 cm^–1^ region corresponds to O–H and N–H stretching vibrations, indicating the presence of hydroxyl and amine groups from chitosan. This broadband remains prominent in both spectra, signifying the consistent presence of hydrogen bonding in the nanofibers, although an increase in intensity is observed in the silver-modified sample, potentially due to interactions between silver nanoparticles and the functional groups of chitosan. The C–H stretching vibrations from the aliphatic chains of PLA are evident in the 2850–2950 cm^–1^ range in both spectra, with no significant changes upon modification with silver nanoparticles, suggesting that the PLA backbone structure remains unaltered.

A sharp peak near 1750 cm^–1^, corresponding to the C=O stretching of ester groups from PLA, is observed in both samples, with no significant shifts or changes in intensity. This indicates that the introduction of silver nanoparticles does not affect the ester carbonyl groups of PLA, preserving the overall structural integrity of the polymer matrix. However, subtle differences are noted in the fingerprint region (1000–1500 cm^–1^), where the C–O stretching and other vibrational modes of chitosan and PLA are detected. In the silver-modified sample, slight modifications in this region suggest some interaction between the silver nanoparticles and the composite matrix, likely through weak coordination or interaction with the chitosan’s hydroxyl and amine groups.

Additionally, the region below 1000 cm^–1^ in the silver-modified nanofibers spectrum exhibits minor changes, which could be attributed to interactions between the silver nanoparticles and functional groups in the nanofibers, potentially forming Ag–O bonds or modifying the local molecular environment. These changes in the lower wavenumber region indicate the successful incorporation of silver nanoparticles into the nanofiber matrix.

### Silver Ions Release

**Figure 4:**
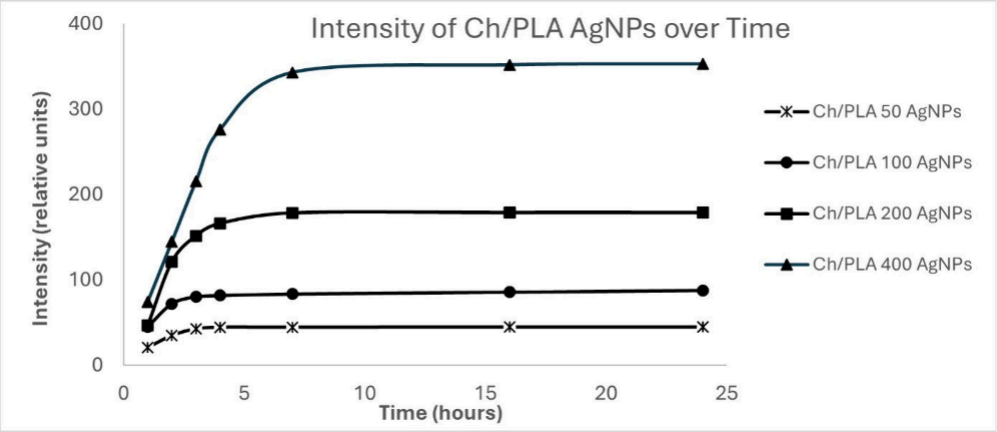
Comparison of the Ag ions release levels of CH/PLA-AgNPs membranes at each time point of experiment for different concentrations (μg/mL).

The experimental results regarding the release of silver ions from nanofibers are illustrated in Figure 4. Based on the shapes of the curves, these results exhibit the typical dynamic characteristics observed in polymer matrices infused with metal ions.^45^

The rapid initial rise in the plotted functions, followed by their stabilization to a plateau, indicates that nearly 90% of the silver was released from the samples within 4 h for all concentration ranges. This also suggests that a significant portion of silver ions were already present in the water within the first few seconds of contact. The shape of the curves demonstrates exceptional repeatability in the experiments and indicates that the ion release process depends on the initial silver concentration. Furthermore, the final amount of silver released exhibited a linear relationship with the total amount of silver in the sample.

### Bacteriological Experiment

Time-dependent bacterial growth assay was conducted on *Staphylococcus aureus (S. aureus)* and *Escherichia coli (E. coli)* to evaluate how bacterial populations change over time in response to various sample applications determining their effectiveness over time. Resazurin assay is effective in quantifying microbial growth without the need for complex sample pretreatments like sonication. This feature is particularly advantageous when working with delicate membranes prone to damage. Resazurin assay allows to quantify the metabolic activity of bacteria, providing insights into how treatment impacts bacterial viability. We recorded color changes over time at different bacterial concentrations to explore the correlation between bacterial concentration and resazurin metabolization rate.

Subsequently, we tailored the incubation times (4 h) to match the growth patterns of the specific species under investigation. Our findings revealed comparable results with the Resazurin assay, indicating a significant antibacterial effect for AgNPs-functionalized membranes and minimal impact for nonfunctionalized ones, likely due to chitosan’s inherent antibacterial activity.

The Resazurin assay underscored the substantial antimicrobial effect of AgNPs-functionalized membranes, particularly evident in samples loaded with 400 μg/mL and 200 μg/mL nanoparticles within all tested periods (SI, Figure S2).

**Figure 5:**
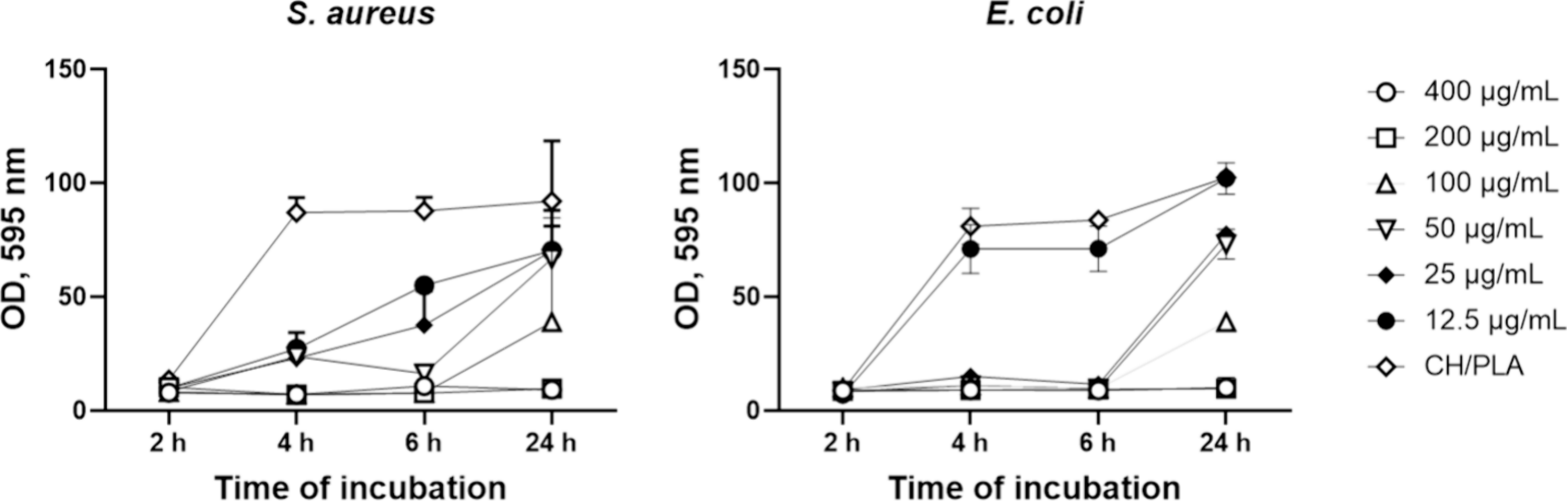
Assessment of the antibacterial properties of CH/PLA-AgNPs membranes against *S. aureus* and *E. coli*. Control: CH/PLA nonloaded membrane, amount of AgNPs loaded to CH/PLA represented in μg/mL.

The heightened antimicrobial activity highlights the potential of AgNP-functionalized membranes for combating microbial infections. However, starting with a concentration of 100 μg/mL tested samples lost their activity after 24 h coincubation (Figure 5). Noticeably, 50 μg/mL and 25 μg/mL of nanoparticles enforced a stronger antibacterial effect on *E. coli* than S. aureus up to 6 h of experiment. Samples incorporated with 12.5 μg/mL AgNPs showed a similar antimicrobial dynamic as nonloaded CH/PLA for *E. coli* but stronger for S. aureus.

### Antiadhesive Activity

**Figure 6:**
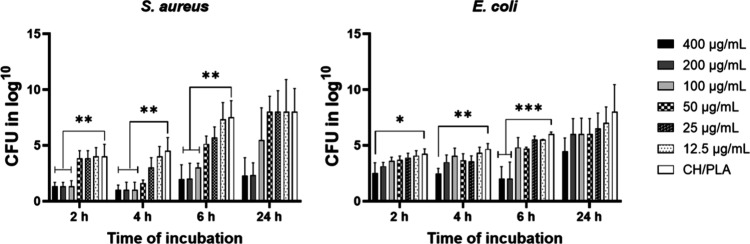
Antiadhesive activity of the CH/PLA nanofiber membranes loaded with silver nanoparticles against *S. aureus* and *E. coli*. CH/PLA indicates the nonloaded membrane, and the amount of AgNPs loaded to CH/PLA is represented in μg/mL.

A significant AgNPs dose-dependent reduction of *S. aureus* and *E. coli* bacterial population was observed up to 6 h point of the assay (Figure 6). Moreover, there was a noticeable time-dependent dynamic of the reduction rate of the total quantity of both bacterial strains incubated with samples doped with 100 μg/mL, 200 μg/mL, and 400 μg/mL of silver nanoparticles against *S. aureus*. Thereby, up to 6 h of cocultivation with membranes containing 100 μg/mL to 400 μg/mL AgNPs, the antiadhesive ability of tested samples was higher toward *S. aureus*. Noticeably, smaller concentrations of doped nanoparticles (50, 25, and 12,5 μg/mL) did not depict a significant difference in antibiofilm activity depending on the type of bacteria.

### Crystal Violet Biofilm Assay

**Figure 7:**
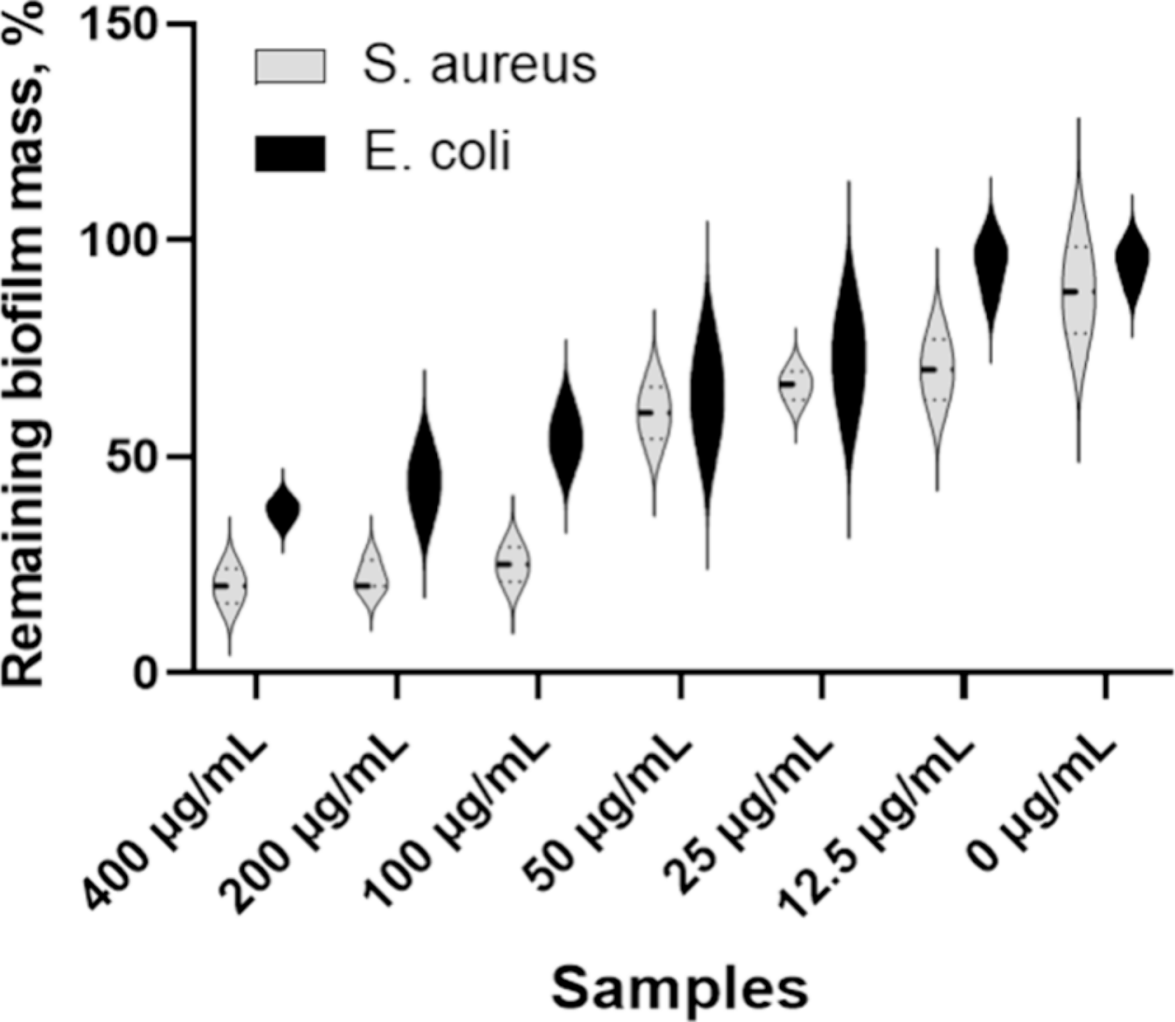
Crystal violet biofilm assay: graphical representation of remaining biofilm mass, %. CH/PLA indicates the nonloaded membrane, and the amount of AgNPs loaded to CH/PLA is represented in μg/mL.

In the context of biofilm formation on CH/PLA membranes with AgNPs, comparing *S. aureus* and *E. coli* can reveal differences in their susceptibility to silver nanoparticles and biofilm-inhibiting materials (Figure 7; SI, Figure S3). Studies generally show that Gram-negative bacteria like *E. coli* are more susceptible to AgNPs than Gram-positive bacteria like *S. aureus*, potentially due to differences in cell wall structure and permeability. In summary, AgNP-loaded CH/PLA membranes are likely more effective against *E. coli* biofilms than *S. aureus*, with *E. coli* showing greater sensitivity to lower AgNP concentrations. However, both bacteria may exhibit significant biofilm inhibition at higher AgNP concentrations.

**Figure 8:**
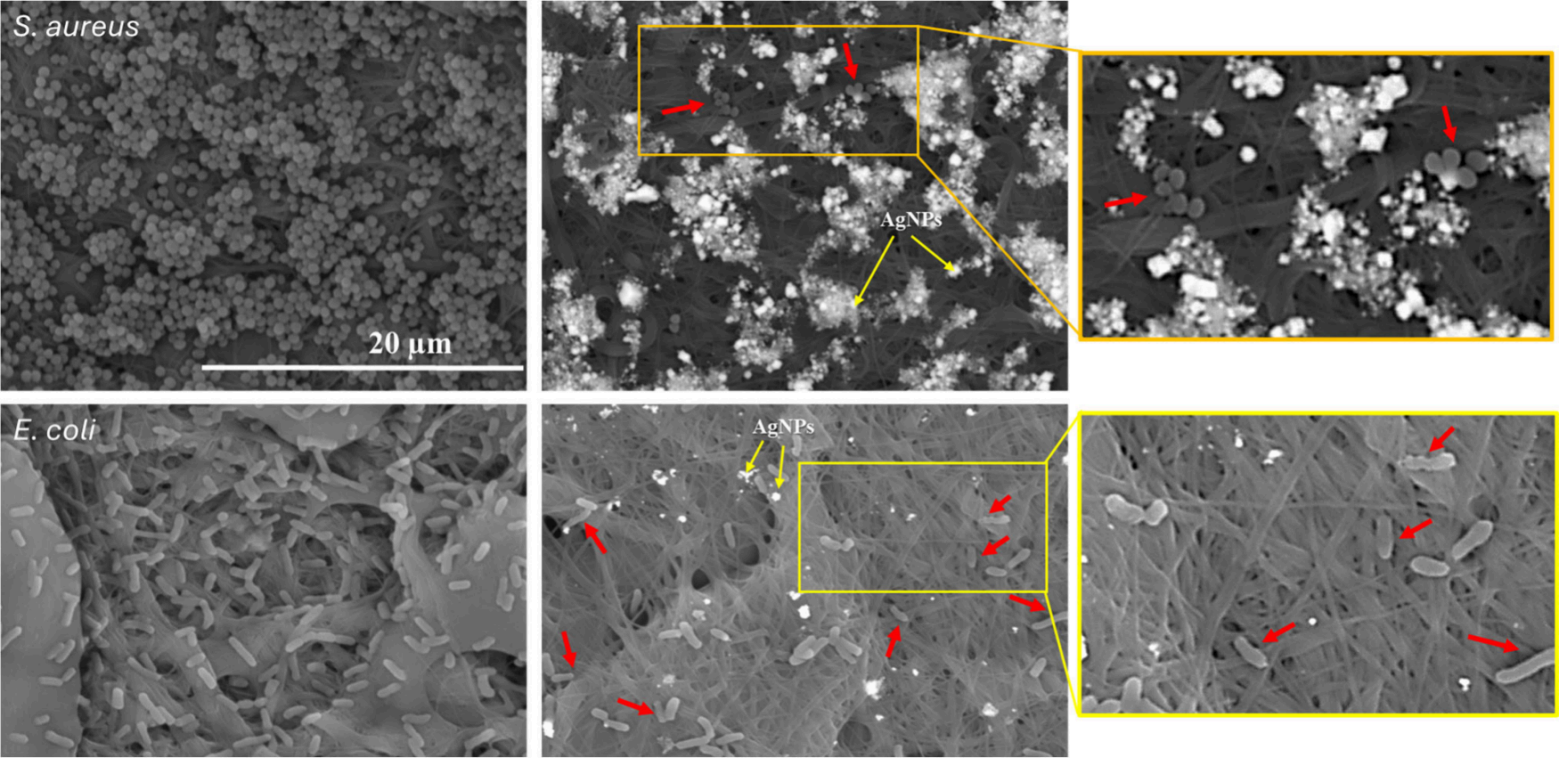
SEM images of the control CH/PLA nanofiber membranes (right column) and loaded with silver nanoparticles CH/PLA 200 μg/mL AgNPs (left column) against *S. aureus* and *E. coli* after 24 h of coincubation.

Biofilm formation study as a qualitative test helped to visualize the efficiency of the fibers as scaffolds against bacterial strains (Figure 8). CH/PLA control membrane did not possess antibacterial properties.^46^ SEM images detected the bacterial biofilm formation along the fibers for both strains. The fiber melting was detected, especially for *E. coli* strains. The observation of fiber melting suggests that bacteria may break down the PLA.^47^ However, adding micro and nanosized chemicals may change and improve PLA qualities. CH/PLA combined with AgNPs showed antibacterial effects via resisting bacterial growth. CH/PLA - 200 μg/mL fibers were chosen to prove the inhibited growth of the biofilm of both strains. There were only small groups of bacteria for *E. coli* strains. Meanwhile, the *S. aureus* bacteria appeared individually. Both types of bacteria underwent several abnormalities in morphology. They presented signs of lysis with their outer layer broken or disrupted. Most of them shrank or changed their form.^48^

### Biocompatibility Assessment

**Figure 9:**
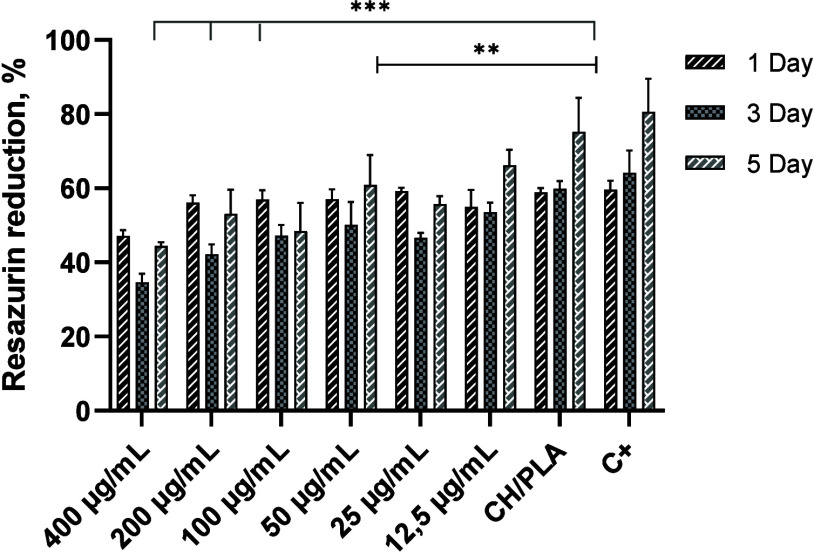
Resazurin reduction assay data on cytotoxicity of CH/PLA membranes immobilized with different concentrations of AgNPs during 5 days of the cell culture experiment. CH/PLA indicates the nonloaded membrane and the amount of AgNPs loaded to CH/PLA is represented in μg/mL. Statistical significance indicated differences between different groups: **p ≤ 0.01, ***p ≤ 0.001.

The results of the resazurin reduction assay after a five-day incubation with UC MSC showed the dependence of cell viability and proliferation activity on the concentration of silver nanoparticles on the CH/PLA membrane (Figure 9). CH/PLA membranes with concentrations of 400, 200, and 100 μg/mL AgNPs showed significant toxic effects on the cells. Despite significant differences with control samples, CH/PLA membrane loaded with 25 and 50 μg/mL of AgPNs demonstrates increasing cell proliferation from day 3 to day 5, which could suggest minor toxicity of these materials. It is important to note that the deposition of silver nanoparticles on CH/PLA membranes at a concentration of 12.5 μg/mL does not show cytotoxic effects and promotes cell proliferation and adhesion. The obtained results demonstrate high biocompatibility and cell proliferation for CH/PLA membranes with a concentration of AgNPs at 12.5 μg/mL.

## Discussion

This study comprehensively investigates the antibacterial properties, adhesive capabilities, and cytotoxicity of chitosan membranes with integrated silver nanoparticles (AgNPs), employing a variety of analytical techniques to provide a robust assessment. Using both classical Petri dish inoculation and the resazurin assay, we evaluated the antibacterial effectiveness of the chitosan membranes.^46^ The resazurin assay, in particular, offered nuanced insights by using a nonfluorescent dye that transitions from blue to pink upon reduction by viable cells, effectively indicating bacterial viability and metabolic activity. This method allowed for detailed quantification of bacterial growth inhibition, providing a complementary perspective to the traditional inoculation method.^48−53^ To overcome limitations inherent in single-method approaches, we incorporated plate counting and crystal violet staining to enhance understanding of microbial biofilm interactions Higher concentrations of AgNPs in chitosan membranes significantly enhanced antibacterial effectiveness, evidenced by reductions in absorbance readings over time, supporting our hypothesis that AgNPs synergize with chitosan to boost antibacterial efficacy, especially against both Gram-positive and Gram-negative bacteria. This multifaceted approach underscores the membrane’s potential as an antibacterial agent and adhesive material, offering comprehensive insight into its application scope.^54^

Our results has demonstrated that chitosan significantly impacts Gram-negative bacteria more than Gram-positive bacteria, attributed to their higher hydrophilicity.^55^ This finding aligns with other studies and highlights the potential of chitosan in targeting Gram-negative strains.^56^ Additionally, silver doping within nanofiber membranes was shown to enhance antibacterial effectiveness across both bacterial categories. The shared antibacterial mechanism between chitosan and AgNPs, involving disruption of membrane integrity and intracellular leakage, underlines their complementary actions.^57^

Chitosan’s antibacterial effectiveness evolves as it degrades over time, releasing short oligomers that exert antimicrobial effects by disrupting bacterial cell membranes, altering permeability, and impeding essential cellular processes. The gradual release of these oligomers allows for sustained antibacterial action, making chitosan a highly suitable candidate for wound care applications.^58,59^

Moreover, the biodegradability and biocompatibility of chitosan further enhance its suitability as an antimicrobial agent and a carrier for drug delivery.^60^ Integrating AgNPs at optimized concentrations (beginning at 50 μg/mL) initially inhibited bacterial growth across both strains, though effectiveness diminished over extended periods, suggesting a need to tailor AgNP concentration for specific clinical requirements.

Optimizing the concentration of silver nanoparticles within dressings is crucial to ensure effective antimicrobial action against bacteria while minimizing potential harm to host cells within the wound. Studies consistently highlight that the toxicity associated with AgNPs primarily stems from the release of silver ions rather than the nanoparticles themselves. However, interpreting results from in vitro experiments requires caution, as they may not fully capture the complex interactions occurring within the wound environment, which comprises multiple cell types and layers.^61^ As demonstrated in this research, Ch/PLA membranes release AgNPs within the first 4 h, significantly impacting cell viability, with a minimally biocompatible concentration of AgNPs below 25 μg/mL. However, in wound conditions, silver ions may be diluted by wound exudate, potentially reducing silver toxicity and allowing for the use of higher concentrations in dressing materials.

The mechanisms of AgNPs’ antibacterial activity may vary with time and concentration. Higher concentrations (e.g., 400 μg/mL) demonstrated robust initial biofilm reduction, though prolonged exposure could lead to biofilm matrix adaptation. At lower AgNP concentrations, bacterial survival and metabolic adaptation influenced biofilm dynamics, with biofilm proliferation resuming after an initial decrease. Incorporating biocompatible materials like chitosan alongside AgNPs not only amplifies antimicrobial action but also moderates potential toxicity, enabling the use of lower AgNP concentrations while achieving effective bacterial inhibition.

Furthermore, AgNP-loaded dressings support wound healing through modulation of metalloproteinase activity, key enzymes involved in tissue repair. AgNPs promote bacterial cell death, reduce inflammation, and regulate cytokine levels, preventing tissue damage. Strategies to control AgNP release or concentration adjustment within dressings can effectively balance antimicrobial action with wound healing and tissue regeneration benefits.

Excessive silver ions in the wound exudate can form inactive compounds, thereby reducing the risk of metal toxicity. Moreover, AgNPs tend to aggregate under physiological conditions, limiting their penetration into deeper skin layers and reducing the likelihood of systemic exposure. Additionally, evidence suggests that silver nanoparticles in dressings predominantly target bacteria near the wound surface, minimizing their impact on deeper tissues.^62−64^

AgNP-loaded dressings expedite wound healing by modulating metalloproteinases’ activity, crucial tissue repair enzymes. AgNPs promote bacterial cell death, alleviate inflammation, and modulate cytokine levels by regulating metalloproteinase levels, thereby preventing tissue damage.^65^

Based on these results, strategies to mitigate the potential toxicity of AgNPs involve controlling their release rate or adjusting their concentration in dressings. Alternatively, incorporating an optimal amount of AgNPs into nanofibrous membranes can ensure effective antibacterial action while promoting wound healing and tissue regeneration.

## Conclusions

This study highlights the significant potential of electrospun chitosan (CH) and polylactic acid (PLA) membranes as antimicrobial coatings for biomedical applications. The combination of CH and PLA results in a porous, hydrophilic structure that supports cell adhesion and proliferation, essential for tissue regeneration. Incorporating silver nanoparticles (AgNPs) at concentrations of 25–50 μg/mL enhances antibacterial efficacy, particularly against *S. aureus* and *E. coli*, and improves antibiofilm activity. Biocompatibility assessments with umbilical cord mesenchymal stem cells revealed dose-dependent toxicity, which may be mitigated in physiological wound environments, suggesting safe and effective usage for biomedical applications. These findings support the potential of electrospun chitosan membranes for wound dressings, tissue engineering, and drug delivery. Future studies should focus on tailoring these membranes for specific wound management applications, carefully balancing antibacterial and cytotoxic effects. Assessing the long-term effects of these nanoparticles in vivo models will be crucial to ensure their safety and efficacy in clinical settings.

## Experimental Section

### Materials

The low-molecular-weight chitosan powder (890 000 Da) was acquired from Glentham Life Sciences in Corsham, United Kingdom (CAS 9012–76–4), while the 1.0 M acetic acid solution (CAS 7732–18–5) was sourced from Honeywell in Charlotte, North Carolina, US. All other reagents, including Poly(l-lactide) powder (average Mn 40,000, CAS 26161–42–2), Poly(ethylene oxide) powder (average Mv ∼ 300,000, CAS 25322–68–3), Polyethylene Glycol (MW 1500, CAS 25322–68–3), chloroform (≥99%, CAS 67–66–3), Ethyl alcohol (≥99.8%, CAS 64–17–5), and NaOH (CAS 1310–73–2), were procured from Sigma-Aldrich in St. Louis, MO, USA.

### Development of Chitosan Membrane

Initially, we mix 10 mL of 99.9% acetic acid (previously dissolved in distilled water to a final concentration of 50% and adjusted to a volume of 20 mL) with 1.6 g of chitosan powder, followed by the addition of 1.6 g of poly(ethylene oxide) (PEO). Subsequently, 0.2 g of polylactic acid (PLA) was dissolved in 5 mL of chloroform, with the removal of excess chloroform. The chitosan solution was then mixed with the dissolved PLA, and 1.2 g of polyethylene glycol (PEG) was added to the solution, following an identical preparation process.

To initiate the electrospinning process, a 50 mL syringe equipped with a needle (inner diameter of 0.69 mm) was filled with the polymer solution. The needle was positioned 15 cm away from the collector, and the electrospinning conditions included a flow rate of 1.5 mL/hour and an applied voltage of 25 kV. Operating conditions encompassed humidity levels below 35% and a temperature range of 21–23 °C. Nanofiber membranes were formed on a 3 cm diameter electrospinning collector, and the resulting chitosan nanofiber membranes were air-dried at room temperature to eliminate solvent residues.

The as-spun Ch/PLA membranes underwent treatment with 1 M sodium hydroxide (NaOH) to reduce their extreme solubility and preserve their nanofibrous structure. The membranes were neutralized using a 1 M NaOH alkali solution (70% ethanol/30% aqueous solution) for 12 h. Subsequently, they were thoroughly washed with distilled water and left to dry overnight at room temperature.

### Decoration of CH/PLA Nanofiber Membranes with AgNPs

The electrospun nanofiber membranes comprised of CH/PLA were subjected to functionalization with silver nanoparticles (AgNPs, Nano Pure Co., Poland). These AgNPs were synthesized within a stainless-steel ultraviolet light reactor and filtered using a reverse osmosis membrane. Characterized by a cubic morphology, the AgNPs employed in this study possessed an average size of 27 ± 4.3 nm.^66^ Incorporation of AgNPs into the CH/PLA membrane was achieved via drop-coating at various concentrations: 12.5 μg/mL, 25 μg/mL, 50 μg/mL, 100 μg/mL, 200 μg/mL, and 400 μg/mL. After coating, samples were air-dried for 24 h at room temperature.

### Scanning Electron Microscopy (SEM) and Contact angle

Scanning electron microscopy (SEM) of the electrospun membrane structure and bacterial colonization were investigated by SEO-SEM Inspect S50–B (FEI, Brno, Czech Republic) with an accelerating voltage of 15 kV, equipped with an energy-dispersive X-ray spectrometer (AZtec One with X-MaxN20, Oxford Instruments plc, Abingdon, UK). The elemental composition of the membrane was detected through EDX analysis. The cross-sectional view of the samples was obtained by cutting samples as follows: membranes were fixed by EM-Tec S-Clip sample holder with 1xS-Clip at a 90° angle to the electron beam. The fiber’s diameter and orientation, and membrane’ local porosity’ (fiber entanglement) were measured using Fiji software (ImageJ 1.51f; Java 1.8.0_102).^67^ ImageJ software was utilized to assess fiber orientation by generating a color-coded map. The local orientation angle ranged from −90° to 90° relative to the horizontal axis. ’Porous area fraction’ was determined using computer binary image analysis. This involved segmenting images into black (porous) and white (substrate) regions via gray-level thresholding. The porous area fraction was calculated as the pore area ratio to the total investigated area. The average values of fiber diameters and ’porous area fraction’ were reported along with their standard deviations. Before analysis, the samples were coated with gold sputter to make them electrically conductive.

The wettability parameter was calculated by measuring the contact angle (CA) be-tween a solid surface and tangent to the wetting agent using a video-based optical contact angle measuring instrument (OCA 15 EC, Data Physics, St. Riverside, CA, USA). CA values were recorded for ultrapure water through at least three parallel measurements. The drop volume value was set at 5 μL. After a drop of liquid has dropped on the electrospun membrane, the straight line is determined as a point corresponding to the contact point between the solid, liquid, and air phases. Wettability values are defined as follows: superhydrophobicity is equal to 0° < θ < 10°, hydrophilicity 10° < θ < 90°, hydrophobic properties 90° < θ < 180° and superhydrophobic θ > 180°.

### FTIR Spectroscopy analysis

The samples were analyzed by Fourier-transform infrared (FTIR) spectroscopy. The measurements were carried out on FT/IR-4700 spectrometer (JASCO) equipped with an ATR PRO ONE module, covering the spectral range of 400–4000 cm^–1^ at room temperature.

### Release of Silver Ions and ICP-MS Analysis

Nanofiber membranes of CH/PLA functionalized with AgNPs (as described in the Silver Ions Release section) were immersed in 50 mL of deionized water (grade 1, EC < 0.055 μS/cm) for release studies. Samples were collected from this solution at regular intervals of 1, 2, 3, 4, 7, 16, and 24 h. The concentration of silver in the collected samples was measured using ICP-MS.

To prepare the samples for analysis, each was acidified with high-purity nitric acid (TraceMetal grade, Fisher Chemical) to achieve a final concentration of 2% (v/v). The acidified samples were then analyzed using the Agilent 8900 ICP-MS QQQ instrument, equipped with a Micromist nebulizer and a helium collision cell. The main instrumental parameters for the ICP-MS analysis are outlined in Table 1.

**Table 1:** Summary of Instrumental Parameters for Inductively Coupled Plasma Mass Spectrometry (ICP-MS)

Parameter	Setting
RF Power (W)	1550
Sampling Depth (mm)	8.0
Plasma Gas Flow Rate (L min^–1^)	15.0
Nebulizer Gas Flow Rate (mL min^–1^)	0.90
He cell gas flow (mL min^–1^)	5.0

To ensure accuracy, the blank correction was utilized, employing ultraclean reagents (TraceMetal grade, Fisher Chemical) and appropriate blanks to monitor and correct any potential background contamination. System stability during measurements was maintained using an internal standard solution (10 μg L^–1^, Agilent). Additionally, for every ten samples, two standard solutions (10 μg L^–1^) were analyzed to confirm system stability and validate the accuracy of the results.

Data processing, collection, and result calculation were conducted using the MassHunter workstation program, which included subprograms for instrument control and offline data analysis.

### Bacteriological Experiment

*E. coli* (ATCC 25922) and *S. aureus* (ATCC 25923) were chosen to assess the antibacterial efficacy of CH/PLA membranes. The bacterial strains were cultured in a nutrient broth at 37 °C for 24 h. Samples (with a surface area of 0.5 cm^2^) were employed for the tests. Prior to the experimentation, all samples underwent sterilization using UV-light radiation at 254 nm for 15 min on each side to ensure sterility and eliminate any potential contaminants. The experiments were performed in three replicates.

### Time-Dependent Bacterial Growth Assay

Tested membranes composed of CH/PLA and loaded with varying concentrations of AgNPs were evaluated for their antibacterial activity. The control sample (nonloaded nanoparticles) and the test membranes were exposed to overnight cultures of *S. aureus* and *E. coli* (10^5^ CFU/mL) for 24 h at 37 °C in 24-well plates. Subsequently, 200 μL of suspension per well was transferred to sterile 96-well plates. Sterile broth served as the negative control, while bacterial broth served as the positive control.

The resazurin assay utilized a 10% v/v concentration of a commercially available resazurin solution (Sigma-Aldrich, USA). The solution was added to each well at 2, 4, 6, and 24 h after coincubation of the samples with bacterial suspension, and absorbances at 570 and 600 nm were measured using a Multiskan SkyHigh Microplate Spectrophotometer (Thermo Fisher Scientific, Waltham, MA, USA) after 4 h of incubation. The percent reduction of resazurin was calculated using the manufacturer’s formula.

### Antiadhesive Activity

Samples were subjected to bacterial broth incubation at 37 °C for 2, 4, 6, and 24 h in 24-well plates. Subsequently, the samples underwent three washes with sterile sodium chloride solution to remove nonadherent cells. To eliminate bacteria adhered to the specimen surfaces, an ultrasonic bath (model B3500S-MT, Branson Ultrasonics Co., Shanghai, China) was used to sonicate the samples in tubes containing 1 mL of sterile saline solution for 1 min.

Following the sonication, 10 μL aliquots of saline solution were inoculated onto nutrient agar plates using the streak plate technique to quantify bacterial count after 24 h of incubation at 37 °C. Control samples, comprising growth medium without bacterial inoculum, were also included.

### Crystal Violet Biofilm Assay

This method allows for high-throughput analysis and is effective for assessing the impact of our samples on biofilm formation and disruption.^68,69^ The biofilm was prepared by inoculating a culture of *E. coli* or *S. aureus* into nutrient broth at concentration of 10^5^ CFU/mL. After this, 1000 μL of the bacterial suspension in MHB medium having 0.5 O.D_600_ was transferred to 24-well plates, where the bacteria were allowed to adhere and form biofilms. This incubation lasted an additional 24 h to enable sufficient biofilm maturation.

After the biofilm was established, the CH/PLA nanofiber membranes, either loaded with silver nanoparticles (AgNPs) or nonloaded, were placed in direct contact with the biofilm. The treatment was conducted at 37 °C for 24 h. Control wells were left untreated with samples. The interaction between the nanofibers and the biofilm was evaluated to assess the disintegration effect through quantitative methods (crystal violet staining), which measured changes in biofilm viability and mass before and after treatment with the nanofibers.

After aspiration of planktonic cells the biofilms were washed three times in 1000 μL of phosphate-buffered saline (PBS). The biofilms were then stained using 1000 μL per well of a 0.1% (w/v) crystal violet solution for 15 min. After that, the excess crystal violet was removed and plates were washed 3 times with 1000 μL of PBS, and air-dried for 30 min. Finally, the cell bound crystal violet was dissolved in 80% ethanol (1000 μL per well). The biofilm biomass was subsequently quantified by absorbance readings at 590 nm (A590) using using a Multiskan FC spectrophotometer (Thermo Fisher Scientific, Waltham, MA, USA). Reduction in biofilm mass ratio (percentage reduction in biofilm mass by comparing the optical density (OD) of wells treated with silver nanoparticles to that of untreated wells) was calculated using Formula 1:
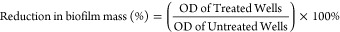
1

After 24 h of incubation in a bacterial suspension, an additional set of samples underwent preparation for SEM study. This aimed to evaluate bacterial cells’ attachment, dissemination, and morphology on nanofibers following a previously described procedure.^70^

### Biocompatibility Assessment

Umbilical cord mesenchymal stem cell culture (UC MSC) was used to assess the cytotoxicity and biocompatibility of the CH/PLA electrospun membranes with different concentrations of AgNPs. A 5 cm segment of the human umbilical cord was obtained from Sumy Regional Clinical Perinatal Center following a normal birth, with the parents’ informed consent, to extract MSCs. This was done in compliance with the ethical clearance granted by the Institutional Review Board of the Medical Institute of Sumy State University’s Commission on Bioethics in Experimental and Clinical Research. As described earlier, Wharton’s jelly was used to obtain the MSC culture. To allow the necessary proteins to adhere to the surface of the membranes, the membranes were placed into a 24-well plate filled with DMEM/F12 medium with 10% fetal bovine serum, 100 units of penicillin, 100 units of streptomycin, and 0.25 mg of Amphotericin B per mL (complete medium) in a humified atmosphere with 5% CO^2^ at 37 °C, and cultured overnight under standard conditions in a cell culture incubator. The following day, 1 mL of whole media was added to each plate containing UC MSC plated at a density of 10,000 cells/cm^2^ (or 34,000 cells per well). Wells containing only cells submerged in the complete medium and wells that contained only the complete medium were used as positive and negative controls respectively.

The cytotoxicity was evaluated by a resazurin reduction assay to evaluate the metabolic capacity of the cultured cells. In order to accomplish this, 15 μg/mL of resazurin was added to the wells, and the cells were then incubated for 8 h under standard conditions. Afterward, 100 μL of the medium were transferred to a second 96-well plate, and the optical density, or absorbance, was determined at 570 and 600 nm using a Multiskan FC plate reader (Thermo Fisher Scientific). A formula from the Method for Measuring Cytotoxicity or Proliferation Using AlamarBlue by Spectrophotometry (BioRad Laboratories) was used to quantify the results.

### Statistics

The results were analyzed statistically using the GraphPad Prism 9.1.1 software package. All experiments were executed in triplicate, and the outcomes are presented as mean ± standard deviation. Significance levels were determined using a one-way analysis of variance (*p* < 0.05 denoting significance).

## Supporting Information Available

Membrane surface wettability, resazurin assay, and crystal violet biofilm assay results (PDF)
