# Supplementary material for: Electrospun Chitosan/Polylactic Acid Nanofibers with Silver Nanoparticles: Structure, Antibacterial, and Cytotoxic Properties
Source: ACS Appl Bio Mater. 2025 Jan 15;8(2):1027–37. doi: 10.1021/acsabm.4c01252 (PMC11836928; doi:10.1021/acsabm.4c01252)
Supplement: Supplementary file 1 — mt4c01252_si_001.pdf [file mt4c01252_si_001.pdf]

ELECTRONIC SUPPORTING INFORMATION

Electrospun Chitosan/Polylactic acid Nanofibers with Silver Nanoparticles: Structure, Antibacterial, and Cytotoxic Properties

Yevhen Samokhin [1], Yuliia Varava [1], Kateryna Diedkova [1,2], Ilya Yanko [1], Valeriia Korniienko [1], Yevheniia Husak [3], Igor Iatsunskyi [4], Vladlens Grebnevs [3,5], Maris Bertiņs [5], Rafal Banasiuk [6], Viktoriia Korniienko [1,2*], Agne Ramanaviciute [7], Maksym Pogorielov [1,2*], Arunas Ramanavicius [7*]

1 Biomedical Research Centre, Sumy State University, 116, Kharkivska, 40007 Sumy, Ukraine; (Y.S.) justinsamokhin@gmail.com; (Y.V.) yuliia.varava@gmail.com; (I.Y) yanko.ilya.brc@gmail.com: (Va.K.) kornienvaleria18@gmail.com
2 Institute of Atomic Physics and Spectroscopy, University of Latvia, Jelgavas iela 3, LV-1004 Riga, Latvia; (M.P.) maksym.pogorielov@lu.lv; (V.K.) viktoriia.korniienko@lu.lv
3 Faculty of Chemistry, Silesian University of Technology, 44-100 Gliwice, Poland; (Y.H.) Yevheniia.Husak@polsl.pl
4 NanoBioMedical Centre, Adam Mickiewicz University, Wszechnicy Piastowskiej 3, 61-614 Poznan, igoyat@amu.edu.pl
5 Faculty of Chemistry, University of Latvia, Jelgavas iela 1, LV-1004 Riga, Latvia; (V.G.) vladlens.grebnevs@lu.lv; (M.B) maris.bertins@lu.lv
6 NanoWave, 02-676 Warsaw, Poland; (R.B.) banasiuk@gmail.com
7 Department of Physical Chemistry, Institute of Chemistry, Faculty of Chemistry and Geosciences, Vilnius University, Naugarduko Str. 24, LT-03225 Vilnius, Lithuania; (Ar.R.) arunas.ramanavicius@chf.vu.lt ; (Ag.R.) agneramanaviciute@gmail.com

* Correspondence: Viktoriia Korniienko, 1,3, viktoriia.korniienko@lu.lv Tel.: +380504071171; Maksym Pogorielov m.pogorielov@gmail.com Tel.: +37122460705; Arunas Ramanavicius arunas.ramanavicius@chf.vu.lt Tel.: +37060032332.

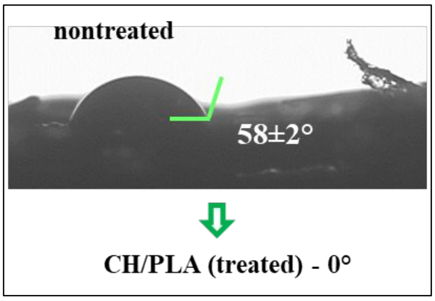

Figure S1: Change in Membrane Surface Wettability Before and After Treatment.

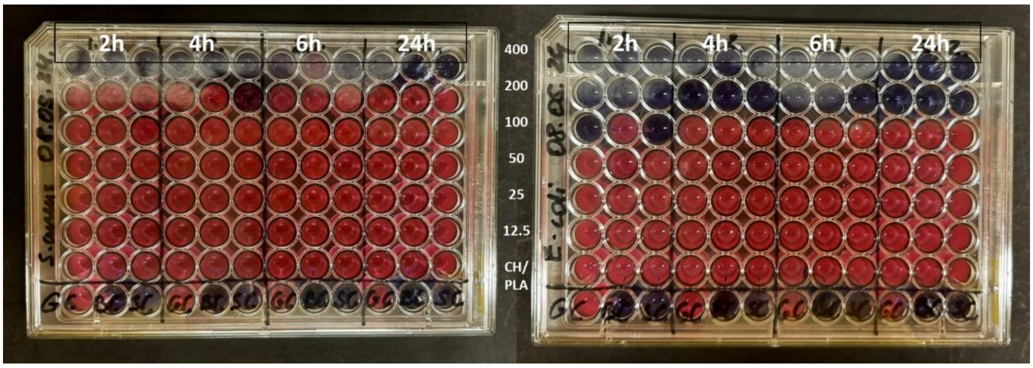

Figure S2: Resazurin assay results. CH/PLA indicates the non-loaded membrane and the amount of AgNPs loaded to CH/PLA is represented in µg/mL. Growth control (GC) – bacterial suspension without tested samples. Broth control (BC) – only growth medium without any bacteria and testedsamples. Sample control (SC) – tested sample with MH broth instead of bacterial suspension.

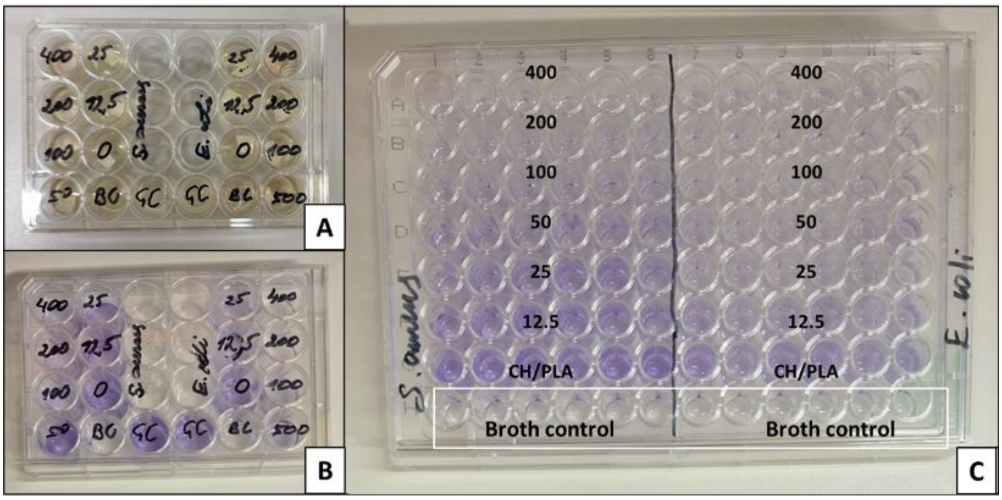

Figure S3: Crystal violet biofilm assay: (A) CH/PLA membranes with biofilm grown for 24 hours in a 24-well plate. (B) 24-well plate after gentian violet staining. (C) absorbance measurements from a 96-well plate, with ethanol extracts transferred from the 24-well plate in six replicates. CH/PLA indicates the non-loaded membrane and the amount of AgNPs loaded to CH/PLA is represented in µg/mL. Growth control (GC) – bacterial suspension without tested samples. Broth control (BC) – only growth medium without any bacteria and testedsamples. Sample control (SC) – tested sample with MH broth instead of bacterial suspension.
